# PRMix: Primary Region Mix Augmentation and Benchmark Dataset for Precise Whole Mouse Brain Anatomical Delineation

**DOI:** 10.1016/j.neuroimage.2026.121881

**Published:** 2026-03-24

**Authors:** Kunhao Yuan, Hanan Woods, Ülkü Günar, Digin Dominic, Ying Wu, Zhen Qiu, Seth G.N. Grant

**Affiliations:** a Institute for Neuroscience and Cardiovascular Research, The University of Edinburgh, UK; b School of Mathematics and Statistics, Yunnan University, China; c Department of Biomedical Engineering, The University of Strathclyde, Glasgow, UK; d Simons Initiative for the Developing Brain (SIDB), Centre for Discovery Brain Sciences, The University of Edinburgh, UK

**Keywords:** Mouse brain delineation, Data augmentation, Fluorescence microscopy, Brain atlas, Dense segmentation

## Abstract

The architecture of the mouse brain shares remarkable similarities with the human brain, making it an essential model for studying brain pathologies, synaptic diversity, and regional specialization. A key step in such studies involves registering molecular images to reference brain atlases, a process hindered by the difficulty of accurately delineating brain regions. Toward this, we have curated a collection of high-resolution, dual-fluorescence microscopy images, termed as dual-fluorescence mouse brain microscopy (DMBM) dataset, complemented by expert annotations of 118 subregions in parasagittal sections. This dataset provides unprecedented insights into the molecular and structural complexity of the mouse brain. However, its full potential for detailed whole-brain analysis is compromised by challenges such as boundary ambiguity and sample scarcity in existing automated segmentation methods, prompting the development of the primary region mix (PRMix) augmentation method. PRMix is specifically designed to expand these datasets, enhance the realism of synthetic data and minimize overlap between adjacent regions. Our approach, together with the curated dataset, achieves superior segmentation performance across the mouse brain compared with existing methods, setting a new benchmark in brain imaging research.

## 1 Introduction

The mouse brain is a fundamental model in neuroscience research owing to its structural and functional parallels with the human brain (Zeisel et al., 2018; Papp et al., 2014). Elucidating its molecular, synaptic, and cellular architecture is essential for understanding brain organization and function. Recent advances in molecular labeling, genetic tagging, and high-resolution imaging have enabled the generation of diverse, brain-wide datasets (Zhu et al., 2018; Bulovaite et al., 2022). However, the standard reference atlases that define brain region boundaries remain based on classical histology (Lein et al., 2007; Paxinos and Watson, 2006), creating a critical need for accurate delineation methods that are compatible with modern molecular imaging modalities (Zhu et al., 2018).

Manual delineation and annotation of brain regions are labor-intensive and prone to inter-annotator variability, limiting both reproducibility and scalability. Automated methods offer a scalable alternative, enabling high-throughput and standardized analyses. However, their performance critically depends on the availability of high-quality and diverse training datasets, which are particularly limited for modalities that integrate molecular information with anatomically accurate annotations. In this context, data augmentation plays a crucial role in enriching datasets by introducing variability and enhancing the robustness and generalization of automated models. Yet, most existing methods focus on a binary segmentation problem, such as tumor segmentation (Menze et al., 2014) or lesion segmentation (Basaran et al., 2023), leaving more complex tasks, such as dense brain delineation, relatively underexplored. To address these challenges, we present the dual-fluorescence mouse brain microscopy (DMBM) dataset, a manually annotated collection of mouse brain parasagittal sections that integrates both structural and molecular information. To improve the precision of automated brain region delineation, we propose primary region mix (PRMix), a novel augmentation method that preserves the original anatomical structure of the brain while minimizing regional overlaps, enabling realistic data synthesis and improving the precision of automated brain delineation (Wang et al., 2022).

## 2 Related work

### 2.1 Brain atlases

The Allen brain atlas (Lein et al., 2007) and Paxinos brain atlases (Paxinos and Watson, 2006) are widely used reference atlases that show delineated regions in planes of tissue sections. A core methodology used to create these atlases is Nissl staining (Kádár et al., 2009), which highlights neuronal cell bodies and cytoarchitecture but lacks the resolution for molecular structures or synaptic connectivity. To overcome this limitation, fluorescent protein probes, such as those fused to endogenous synaptic proteins (Zhu et al., 2018), self-labeling tags such as HaloTags (Los et al., 2008), and antibody labeling (Curran et al., 2021), offer enhanced molecular insight and have been instrumental in developing single-synapse resolution maps of the mouse and human brain. In contrast to the cellular-level view of Nissl staining, protein-marked imaging provides superior subcellular detail.

More recently, advances in high-throughput 3D imaging have accelerated the creation of brain-wide datasets with cellular or subcellular resolution. Light-sheet fluorescence microscopy (LSFM), for instance, has been instrumental in generating whole-brain maps by enabling rapid optical sectioning of cleared tissue (Perens et al., 2021). Similarly, serial two-photon tomography (STPT) has been employed to systematically image and reconstruct the entire mouse brain at micron-scale resolution, providing detailed cytoarchitectural and connectivity data (Vousden et al., 2015).

While these methods provide invaluable insights into whole-brain architecture and function, they often rely on single molecular markers or are optimized for tracing long-range projections.

### 2.2 Dual-synaptic markers

Our work leverages a dual-synaptic marker strategy to dissect the molecular diversity of synapses. The two proteins we visualize, post-synaptic density protein 95 (PSD95) and synapse-associated protein 102 (SAP102), are key scaffolding molecules within the postsynaptic terminal of excitatory synapses but exhibit distinct expression patterns and functional roles throughout development (Metzbower et al., 2024) and across different brain regions (Zhu et al., 2018; Cizeron et al., 2020; Migaud et al., 1998; Cuthbert et al., 2007). Imaging these molecules at single-synapse resolution across the brain has allowed for the generation of the first brain-wide synaptic maps in mammals across the lifespan (Cizeron et al., 2020; Bulovaite et al., 2022), in genetic models of neurodevelopmental disorders (Zhu et al., 2018; Tomas-Roca et al., 2022) and in sleep deprivation (Koukaroudi et al., 2024).

The use of such multi-marker synaptic atlases has vast potential. They provide a crucial baseline for studying how synaptic composition is altered in models of disease, the effect of experience and learning, the impact of pharmacological interventions, and many other applications. Furthermore, this approach can be extended to include additional synaptic markers to create even more detailed synaptome maps (Zhu et al., 2018), enabling researchers to undertake advanced functional (Velicky et al., 2023) and connectomic (Winding et al., 2023) studies.

### 2.3 Image augmentation

Deep learning-based image analysis, especially in medical imaging, often faces data scarcity (Litjens et al., 2017; Frid-Adar et al., 2018). To mitigate this, image augmentation techniques, which were originally popularized in natural image recognition, have been widely adopted. Traditional approaches apply geometric transformations such as flipping and affine adjustments (rotation, scaling, translation) to teach models spatial invariance, as well as intensity perturbations (brightness and contrast shifts) to ensure robustness to variations in imaging conditions (Krizhevsky et al., 2012; Shorten and Khoshgoftaar, 2019; Chen et al., 2020).

More sophisticated techniques have emerged that mix information between images to expand dataset diversity. MixUp (Zhang et al., 2018) introduced linear image combinations to expand datasets and regularize training, and was later extended to segmentation tasks (Ghiasi et al., 2021). Recent advances include patch-based augmentation (Yun et al., 2019), scribble-based methods for medical imaging (Zhang and Zhuang, 2022), semantic-aware augmentation (Zhang et al., 2023; Wang et al., 2025), and self-adaptive blending to address background inconsistencies (Zhu et al., 2022). While effective for object-centric tasks, these methods are nevertheless ill-suited for whole-brain analysis as they disregard global anatomical topology.

### 2.4 Image synthesis

A related, yet distinct, line of research employs generative models to synthesize medical images (Zhu et al., 2017), with a primary focus on modality imputation (*i.e*., translating images between modalities). This has proven effective in applications ranging from magnetic resonance imaging (MRI) and computed tomography (CT) image synthesis (Chartsias et al., 2019; Reaungamornrat et al., 2022), to more recently, in multi-modal brain MRI to reduce data collection requirements (Yu et al., 2022). However, it was not designed for *de novo* sample synthesis and has not demonstrated success on high-resolution microscopy brain images, where cellular and regional integrity is a critical requirement.

PRMix, with its primary region sampling and overlap-aware augmentation, is specifically designed to address these gaps.

## 3 The dual-fluorescence mouse brain microscopy dataset

### 3.1 Data collection

The dataset comprises whole-brain parasagittal sections from 96 mice (48 male, 48 female) ranging in age from 1–12 months. Detailed procedures for the generation of the mouse lines are described in Zhu et al. (2018). Briefly, the endogenous *Psd95* and *Sap102* genes were genetically modified by inserting the coding regions for fluorescent proteins *eGFP* and *mKO2* into the 3’ regions of the genes, resulting in the expression of *PSD95^eGFP^* and *SAP102^mKO2^* fusion proteins. Mice were perfused with sodium pentobarbital and saline-PFA. Brains were dissected, post-fixed, and cryo-embedded in OCT compound. Parasagittal sections (18 μm) were cut referencing Allen mouse brain atlas slices 11–12 (Lein et al., 2007). Whole-brain imaging was performed on a Nikon Eclipse Ti2 with a spinning disk confocal system, and 856 × 812 individual tiles (per image) were stitched into full images with 16× downsampling. Subregions were manually delineated in ImageJ using PSD95/SAP102 protein markers, guided by the Allen brain atlas.

### 3.2 Dataset statistics

**Fig. 1:**
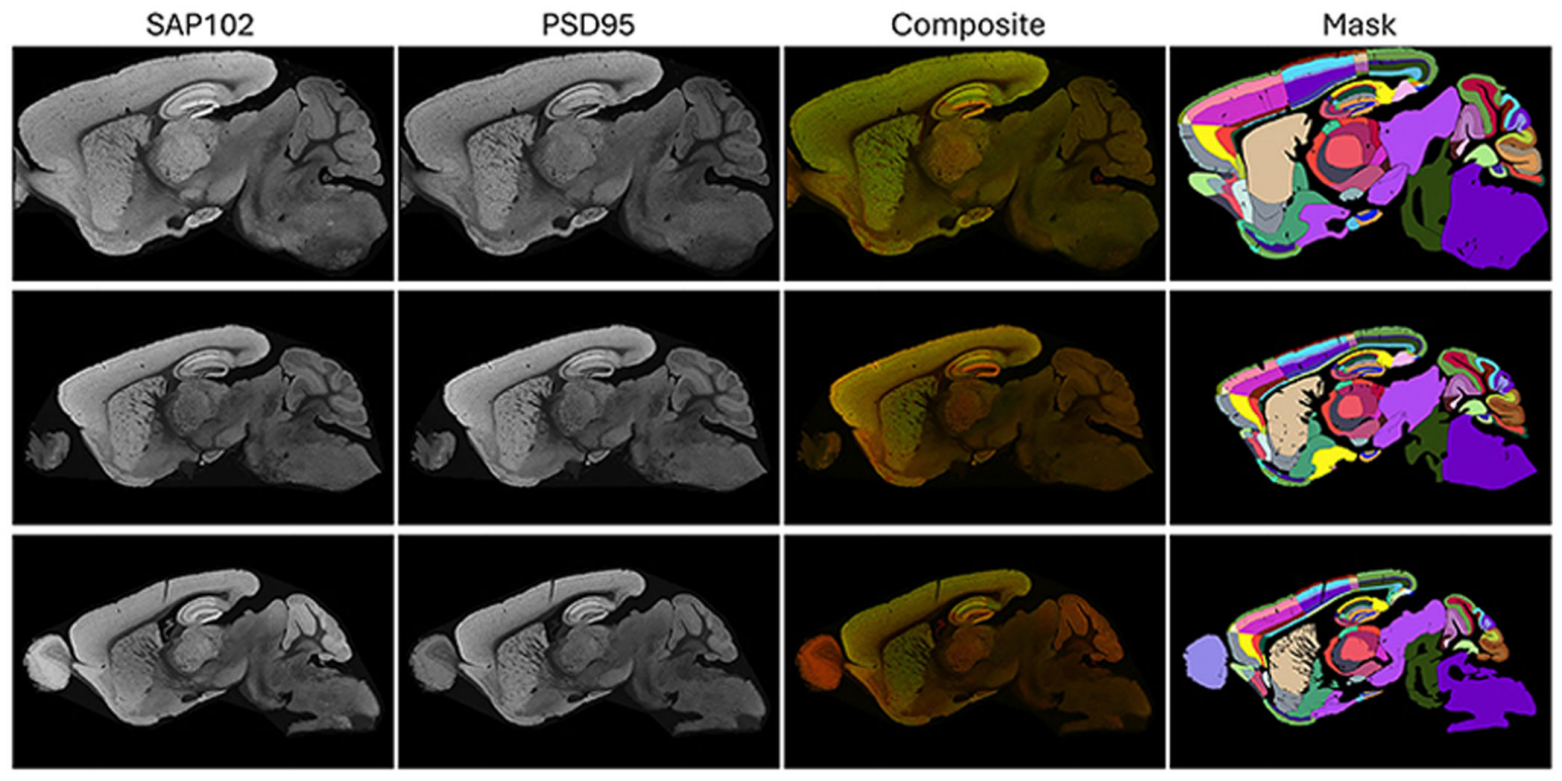
Exemplar images from the curated dataset. From left to right: whole-brain images stained for SAP102 and PSD95, the dual-channel composite image (SAP102 in red, PSD95 in green), and the corresponding manually annotated brain regions.

**Fig. 2:**
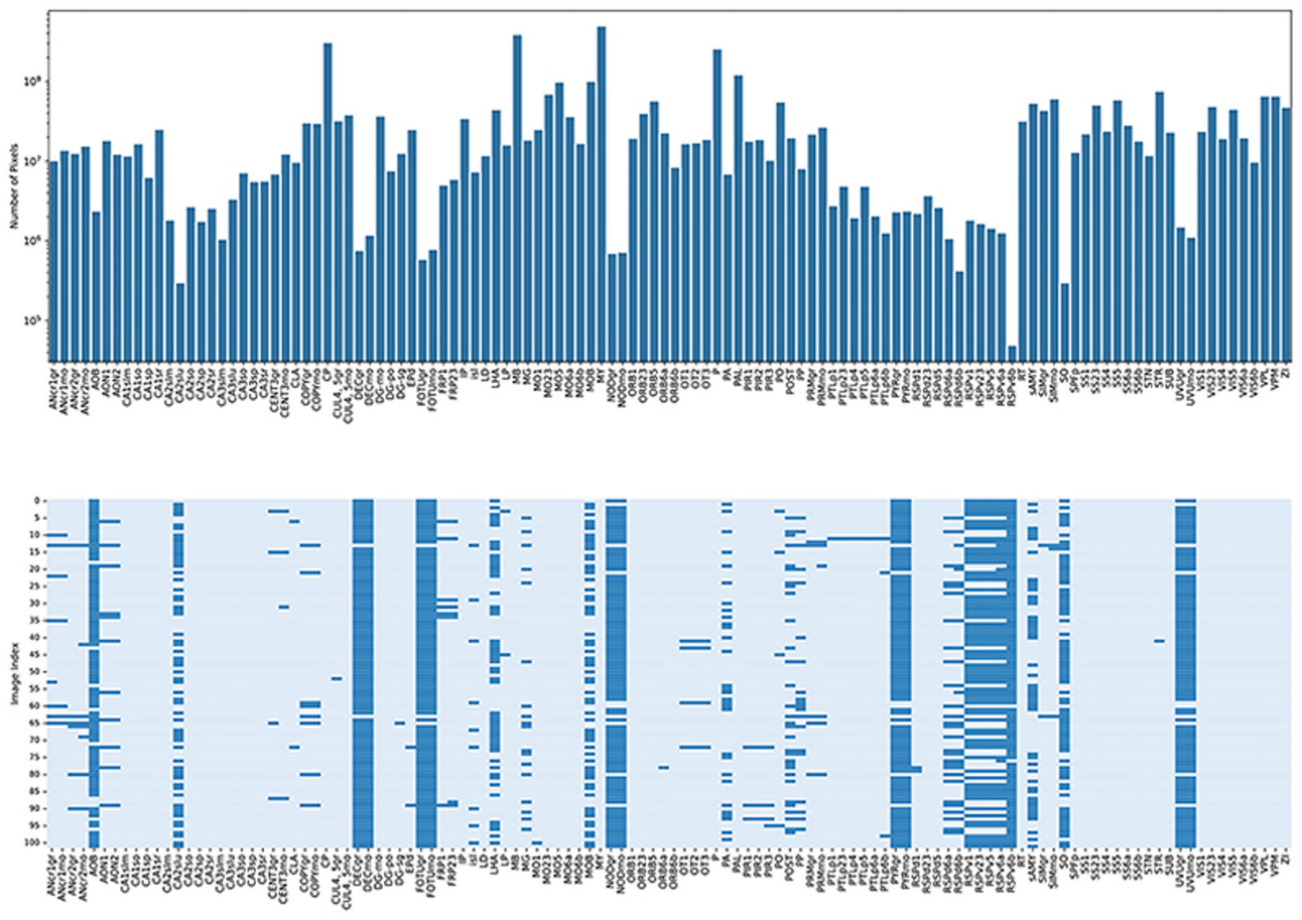
Top (a). Distribution of pixel counts across regions. Bottom (b). Region availability per image with light blue indicating presence and dark blue representing absence.

The collected dataset has **102** dual-fluorescence whole-brain parasagittal images (*n* = 96), with a median resolution of 6383 × 12531, encompassing **118** well-defined anatomical subregions from the sagittal mouse brain slices. Three exemplar images are shown in Fig. 1. The total number of pixels for each subregion is log-rescaled and summarized in Fig. 2(a), which highlights significant variations in pixel counts across subregions. Notably, the foreground-to-background ratio is approximately **0.84**, making the proposed dataset highly challenging while informative for neuroscience and medical research. Fig. 2(b) illustrates the missing subregions for specific image IDs. Due to the location of the slices during sectioning, certain areas, such as AOB and RSPv6b, are present in only two slices and are considered less reliable and representative. We thus excluded them in evaluation but kept the information in the figure for completeness.

## 4 Methods

### 4.1 Motivation

While we introduced a high-resolution, dual-fluorescence dataset, microscopy data are scarcer than for CT (Wasserthal et al., 2023) or MRI (Menze et al., 2014) due to their extremely large size (e.g., 102k× 200k pixels) and low throughput, and thus insufficiently diverse on their own to eliminate augmentation. This scarcity, coupled with biological variability, makes augmentation essential.

**Fig. 3:**
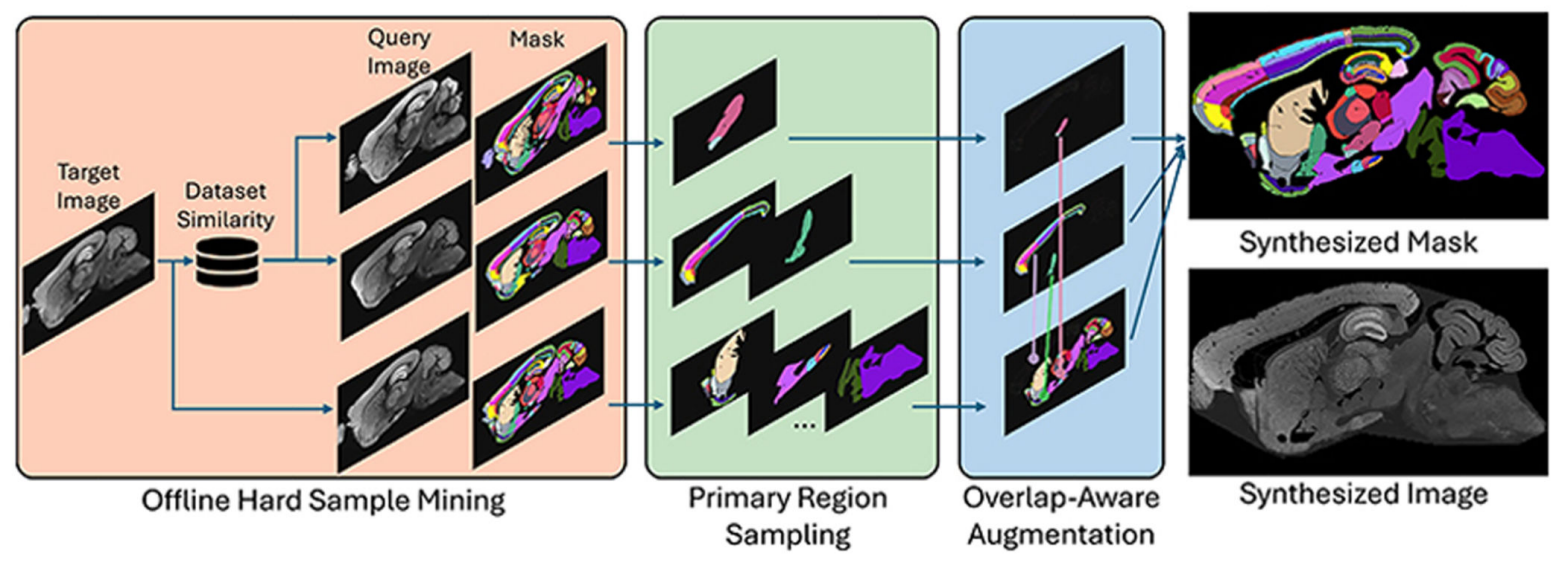
Overview of the proposed PRMix, consisting of three modules (1) offline hard-sample mining (HSM), (2) primary region sampling (PRS) and (3) overlapaware augmentation (OAA). Only a single fluorescent marker is shown for clarity.

Mixing-based augmentations like CutMix (Yun et al., 2019) and CarveMix (Zhang et al., 2023) are suboptimal for our task as they were designed for object-centric tasks and fail to preserve global context. Generative methods such as MouseGAN series (Yu et al., 2022) perform well when synthesizing low-resolution (~0.1 mm) images with a small number of regions (<30 per hemisphere). When applied to high-resolution brain images (~100 nm scale) with over a hundred subregions, they often generate anatomically plausible but texturally blurry structures, which compromises the crucial topological relationships among numerous small subregions. PRMix is thus motivated to enhance data diversity and realism for effective modeling. The overall diagram of the proposed PRMix is illustrated in Fig. 3, with the subsequent paragraphs detailing its design objectives and implementation.

### 4.2 Offline hard-sample mining

**Fig. 4:**
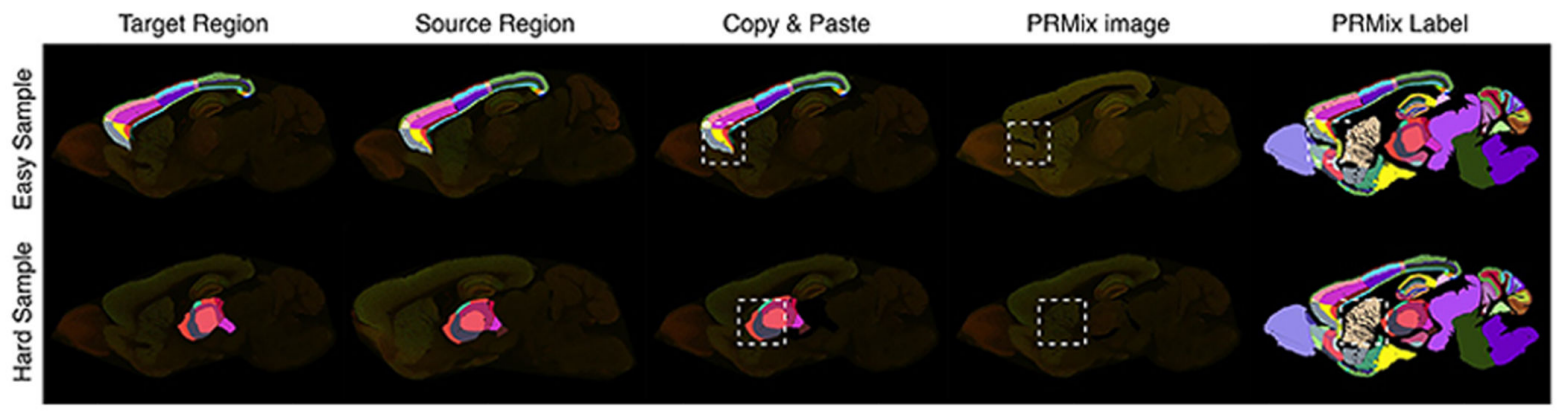
Exemplar samples from the ‘easy’ and ‘hard’ mixing categories. Target primary regions are highlighted with label masks, and affected areas are shown within dashed rectangles. For clarity, only one primary region is selected in the example; however, PRMix can handle mixing multiple primary regions from different sources.

According to our initial experiments, random sample mixing can introduce feature inconsistencies, while exclusively selecting visually similar samples lacks sufficient challenge for effective learning. We therefore designed a strategy to ensure each target image is paired with multiple query images from a diverse range of pre-computed similarities across the whole dataset, termed as offline hard-sample mining. We quantify similarity through a mask overlap score, a metric designed to emphasize structural variations, which constitute the primary challenge in delineation, whereas standard augmentations compensate for variations in texture and intensity. For each target image, similarity scores are computed against the remaining training images and sorted. The top 20% most similar samples are designated as ‘easy’ samples, while the remaining 80% are labeled as ‘hard’ samples. As shown in Fig. 4, ‘easy’ sample-mixing minimally affects adjacent regions. In the ‘hard’ case, however, a direct ‘copy-and-paste’ approach leads to significant overlaps with the striatum and pallidum regions. PRMix resolves this by recalculating the target primary region’s optimal size, orientation, and location to fit properly within the target image. During data augmentation, we mix ‘easy’ and ‘hard’ samples in varying proportions, aiming to find a balance between challenge and learnability. Empirically, oversampling 80% from the hard-sample set yielded the best results. Details of the different sampling ratios are summarized in Table 5.

### 4.3 Primary region sampling

**Fig. 5:**
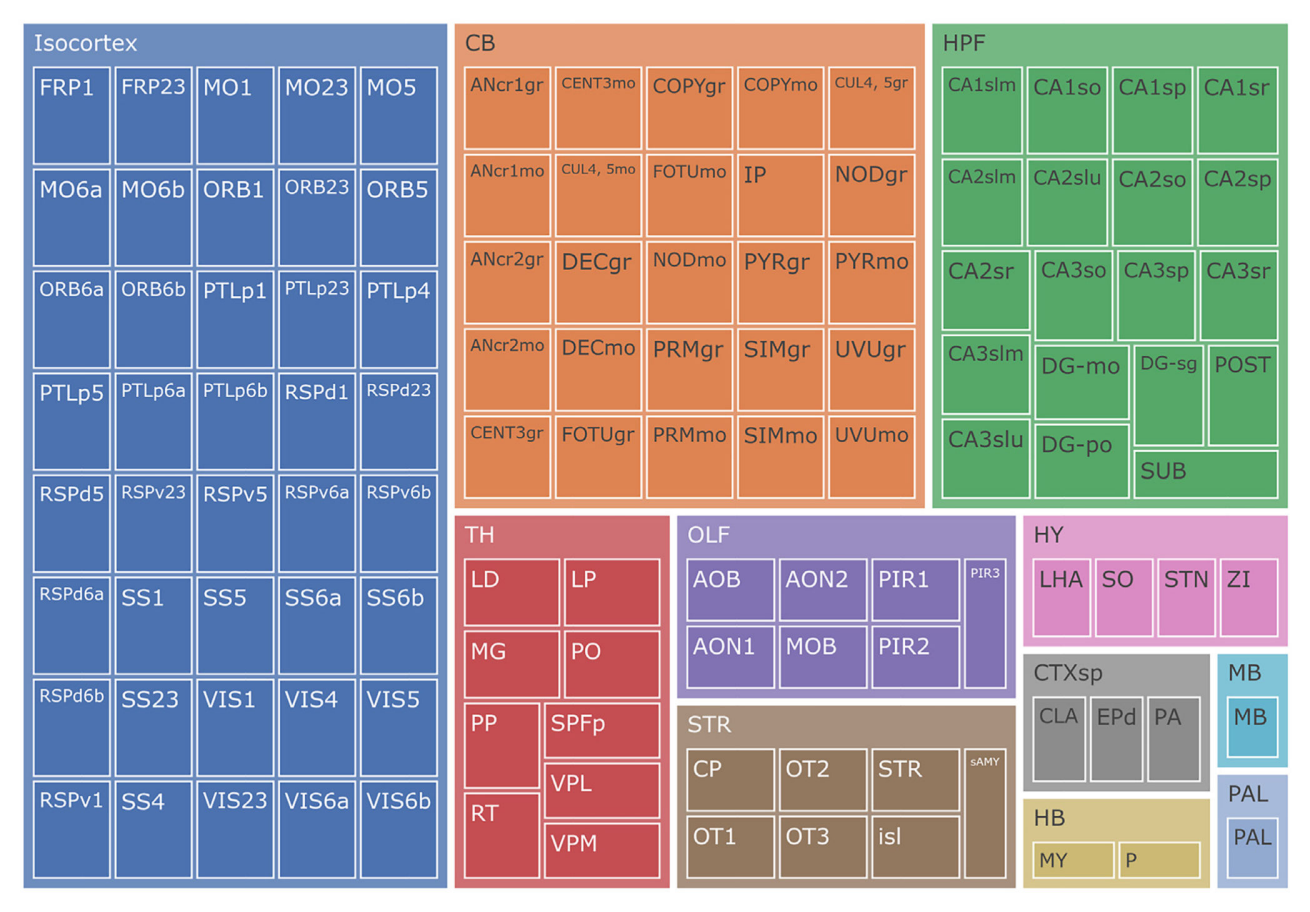
The 11 primary brain regions illustrated in distinct colors, showing the grouping of their 118 constituent subregions.

Based on anatomical and functional criteria (Lein et al., 2007; Papp et al., 2014), we grouped the 118 brain subregions into 11 primary regions: cerebellum (CB), thalamus (TH), midbrain (MB), hindbrain (HB), isocortex, hypothalamus (HY), olfactory areas (OLF), cortical subplate (CTXsp), striatum (STR), pallidum (PAL), and hippocampal formation (HPF), as illustrated in Fig. 5. These primary regions serve as the fundamental units for mixing. During mixing, we only sampled 3–9 of these regions from multiple sources to replace those in target image, boosting diversity while preserving feature consistency. The primary region sampling offers two key advantages: (1) preserving the anatomical structure of the brain image and (2) avoiding potential subregional overlaps during the mixing process. More importantly, our design incorporates mixing at an intermediate semantic level, setting it apart from existing object-centric approaches (Zhang et al., 2018; Yun et al., 2019; Zhang et al., 2023; Wang et al., 2025). This makes it particularly well-suited for dense foreground tasks such as whole-brain delineation.

### 4.4 Overlap-aware augmentation

To ensure those sampled regions align in location and size with the target image, we implemented an overlap-aware augmentation module. This module begins by applying random affine transformations — such as rescaling, rotation, and translation — to the query masks for individual primary regions. Applying these transformations independently to local regions introduces non-linear structural variations, simulating complex geometric distortions — such as local stretching and tissue shifting — frequently observed in histological preparations. Corresponding regions are then cut out from the target masks, leaving behind a complementary mask which is used to calculate intersections with the query mask. If the intersection is below a certain threshold *τ*, the query region will be pasted directly onto the complementary masks at its original location. Otherwise, a greedy search for optimal affine parameters will begin to ensure a minimum intersection where possible. Once the greedy search converges, the optimized affine transformations are reapplied to the query images and masks, followed by the final pasting process. Crucially, this pasting operation is performed synchronously across both fluorescent channels. By transferring the multi-channel signal as a coupled unit, PRMix strictly preserves the intra-region synaptic co-localization patterns — essential for defining molecular identity — even as the background context changes. Further-more, by training the model to resolve anatomical structures despite these local geometric shifts and the artificial boundary discontinuities introduced by mixing, our approach effectively enhances robustness against physical artifacts like tears and folding. The overall process is outlined as pseudo-code in Algorithm 1.

Algorithm 1 **OAA: Overlap-Aware Augmentation**

**input :** Source image, seg. mask, primary region $p:{\mathrm{\{X,Y\}}}_{s}^{p}$; Target seg. mask, primary region $p:Y_{t}^{p}$; Overlap tolerance *τ*; Rot., Scaling, Trans., Shift ranges: $\left[\sigma_{l}^{r},\sigma_{h}^{r}\right],[\sigma_{l}^{s},\sigma_{h}^{s}],\left[\sigma_{l}^{t},\sigma_{h}^{t}\right],\left[s_{l},s_{h}\right]$

**output:** Augmented low-overlap source mask and image: ${\tilde{M}}_{s}^{p},{\tilde{X}}_{s}^{p}$; obtain binary region masks: $M_{s}^{p}\leftarrow 𝕀\left(Y_{s}==p\right),M_{t}^{p}\leftarrow 𝕀\left(Y_{t}==p\right)$; Sample random affine transformations: $T_{r}\sim U\left(\sigma_{l}^{r},\sigma_{h}^{r}\right),T_{s}\sim U\left(\sigma_{l}^{s},\sigma_{h}^{s}\right),T_{t}\sim U\left(\sigma_{l}^{t},\sigma_{h}^{t}\right),T_{\text{aff}}=T_{r}\circ T_{s}\circ T_{t}$; Apply affine transformations to mask: $M_{s}^{p}\leftarrow T_{\text{aff}}\left(M_{s}^{p}\right)$; Initialize best overlaps and shifts: *O* ← +∞ *s_x_, s_y_* ← (*s_I_, s_I_*);

**while** *s_x_* ∈ [*s_I_, s_h_*] **do**

       **while***s_y_* ∈ [*s_I_, s_h_*] **do**

           Shift binary region mask: $M_{\text{tmp}}=T_{\text{shift}}\left(M_{s}^{p},s_{x},s_{y}\right)$;

           Calculate overlaps between the binary mask and the

             complementary target mask: $o=M_{\text{tmp}}\cap \left(1-M_{t}^{p}\right)$;

            **if** *o* < *O* **then**

               *O* ← *o*;

                **break inner loop** if *O* < *τ*;

           **end**

           *s_y_* ← *s_y_* + stepsize;

       **end**

       *s_x_* ← *s_x_* + stepsize;

       **break outer loop** if *O* < *τ*;

**end**

Obtain optimal mask and image: ${\tilde{M}}_{s}^{p}\leftarrow M_{\text{tmp}}$;

  ${\tilde{X}}_{s}^{p}=T_{\text{shift}}\left(X_{s}^{p},s_{x},s_{y}\right)$

### 4.5 Comparison with existing mixing approaches

Before formalizing the proposed PRMix, we compare it with existing mixing-based augmentations. Starting with the simplest method, MixUp (Zhang et al., 2018) blends a pair of images, *i.e*. source image *X_s_* and target image *X_t_* and their corresponding labels *Y_s_* and *Y_t_*. The synthesized image and label are generated through:

(1) $$\begin{array}{l} \tilde{X}=X_{s}\cdot \lambda +X_{t}\cdot (1-\lambda )\\ \tilde{Y}=Y_{s}\cdot \lambda +Y_{t}\cdot (1-\lambda ), \end{array}$$

where *λ* ~ *U*(0, 1) is a controlling factor. For CutMix (Yun et al., 2019) and CarveMix (Zhang et al., 2023), their mixing strategies can be summarized as:

(2) $$\begin{array}{l} \tilde{X}=X_{s}⊙M_{s}+X_{t}⊙\left(1-M_{s}\right)\\ \tilde{Y}=Y_{s}⊙M_{s}+Y_{t}⊙\left(1-M_{s}\right). \end{array}$$

⊙ is the element-wise dot product and *M_s_* represents a binary mask used to sample areas from the source image, which can be either rectangular regions (CutMix) or semantic regions (CarveMix). Taking both source image and target image semantics into account, we yielded the basic form of our PRMix:

(3) $$\begin{array}{l} {\tilde{X}}^{p}={\hat{X}}_{s}^{p}⊙{\hat{M}}_{s}^{p}+X_{t}^{p}⊙\left(1-M_{t}^{p}\right)\\ {\tilde{Y}}^{p}={\hat{Y}}_{s}^{p}⊙{\hat{M}}_{s}^{p}+Y_{t}^{p}⊙\left(1-M_{t}^{p}\right), \end{array}$$

where ${\hat{M}}_{s}^{p}$ is the overlap-mitigated semantic mask for primary region *p*, retrieved with ${\hat{M}}_{s}^{p}=OAA\left(M_{s}^{p},M_{t}^{p}\right),M_{s}^{p}$ is the union of total *k* subregions within a primary region, known as $M_{s}^{p}=M_{s}^{1}\cup M_{s}^{2}\cup \ldots \cup M_{s}^{k}$, and the adapted source image ${\hat{X}}_{s}^{p}$ is generate with the overlap-mitigated semantic mask, using ${\hat{X}}_{s}^{p}=\text{T}\left(X_{s}^{p},{\hat{M}}_{s}^{p}\right)$. Additionally, to enhance the versatility and variability of the dataset, we further extended the proposed PRMix to multiple source images, simply by denoting the above process as ${\tilde{X}}^{p},{\tilde{Y}}^{p}=\text{PRM}\left({\left\{X,Y\right\}}_{s}^{p},{\left\{X,Y\right\}}_{t}^{p}\right),$, and iterated the process multiple times via:

(4) $$\tilde{X},\tilde{Y}=\underset{P\;\text{total}\;\text{number}\;\text{of}\;\text{primary}\;\text{regions}\;}{\underset{︸}{\text{PRM}(\ldots ,(\text{PRM}({\left\{X,Y\right\}}_{s}^{2},\text{PRM}({\left\{X,Y\right\}}_{s}^{1},{\left\{X,Y\right\}}_{t}^{1}))))}}$$

This enables mixing at an intermediate semantic level and allows generalization beyond a single image pair, unlike traditional augmentation methods where repeated lower-level mixing often leads to occlusion and ambiguity.

**Fig. 6:**
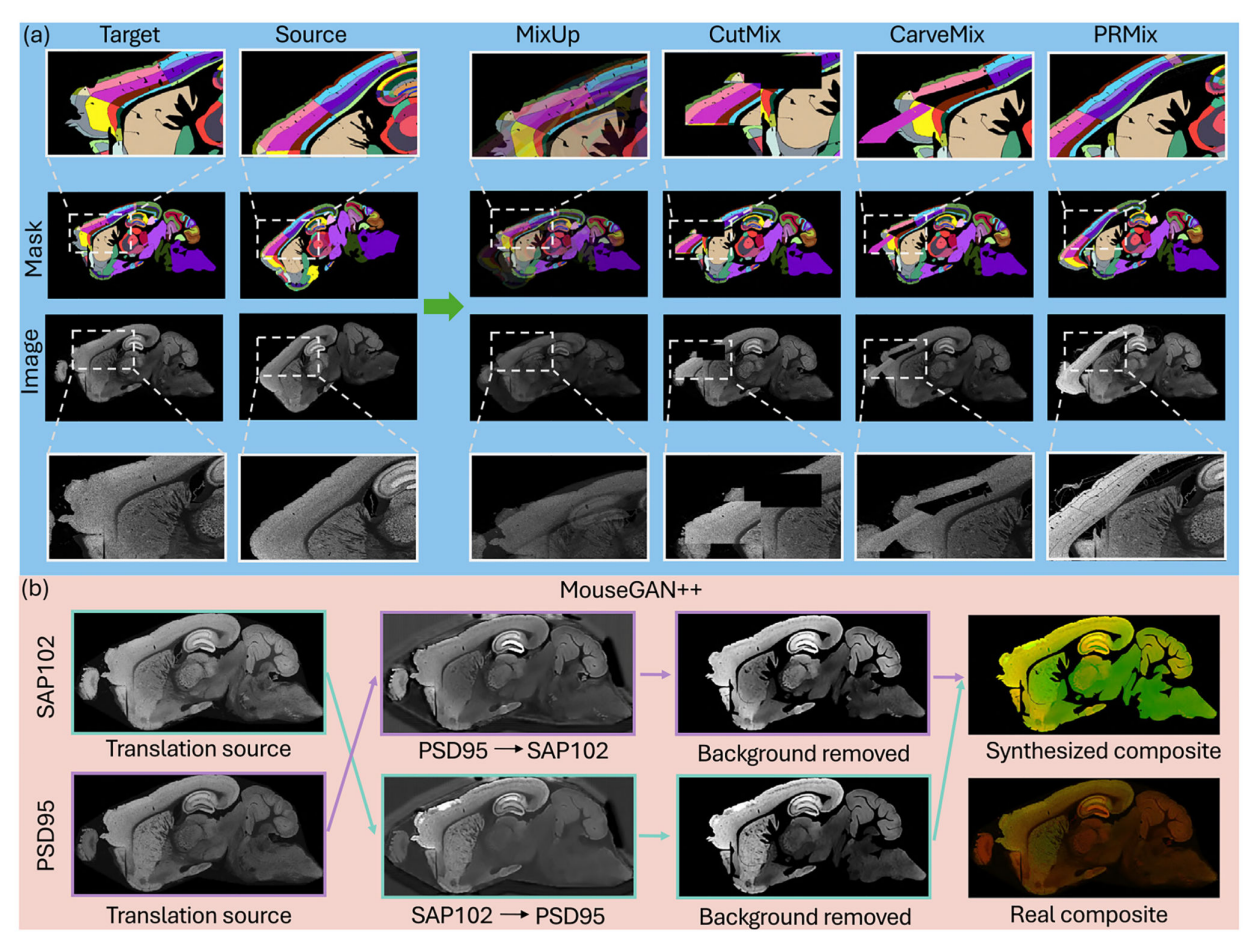
Exemplar results illustrating different augmentation outputs derived from identical source-target pairs. Panel (a): The middle two rows show the original image and its corresponding label mask, while the top and bottom rows provide zoomed-in views of the manipulated regions. Panel (b): The sample synthesis flow for generative method MouseGAN++. Best viewed in color.

Exemplar synthesized images from different methods are illustrated in Fig. 6. MixUp (Zhang et al., 2018) generates ambiguous images, whereas CutMix (Yun et al., 2019) and CarveMix (Zhang et al., 2023) focus on single regions and struggle to preserve global anatomy. By contrast, our method produces the most realistic and diverse-looking images. We also compared our approach to a generative model, MouseGAN++ (Yu et al., 2022), initially designed for modality imputation in MRI. As shown at the bottom of the figure, while this GAN-based method maintains global structure, it does so at the cost of local texture and intensity details, which are essential for dense whole-brain delineation.

## 5 Experiments

### 5.1 Implementation details

To ensure a fair comparison between different augmentation methods and minimize the influence of model architectures, all experiments were conducted using the state-of-the-art medical image segmentation model nnUNet (Isensee et al., 2024), which enhances the classic UNet (Ronneberger et al., 2015) with task-specific configurations. Both the proposed PRMix and its comparators were applied offline to generate augmented datasets for each fold, thereby enhancing training efficiency. Additionally, a shared pipeline was applied during mixing, which included standard pre-processing (intensity normalization, foreground oversampling, color jittering, and affine transformations) and a final morphological opening step after mixing to smooth artificial edges resulting from manual delineation. The model was trained with an input patch size of 768 × 1536, approximately 1/64 of the median image size, using 2 patches per GPU and 250 minibatches per epoch. The collected dataset was split into 80 training/validation and 22 strictly isolated and unaugmented testing samples, and all experiments were conducted using 5-fold cross-validation on two NVIDIA RTX 4090 GPUs.

The augmented training sets were generated with varying folds, namely 5× (~400 images), 10× (~800 images) and 20× (~1600 images), by excluding cases where source regions are absent from the target image. Although MouseGAN++ (Yu et al., 2022) was developed for modality imputation rather than data augmentation, we generated a 20× augmented dataset by repeated random sampling from the generative model. Due to the large size of the whole-brain image, patch-wise training has been employed. Under the given patch size and batch settings, the model requires approximately 2500 iterations to process the entire training dataset once—a process that takes about two days on a single GPU. Consequently, the baseline model was trained for 12 full epochs as a reference, while the proposed PRMix model was evaluated with 3, 6, and 12 full epochs for comparison. Hereafter, unless otherwise specified, all mentions of “epochs” refer to full epochs.

### 5.2 Experimental results

We compared PRMix with existing methods in Table 1, evaluating categorical average precision, recall, F1 and mIoU. Unless stated otherwise, all results are the average of 5-fold runs and were obtained using purely synthesized data (except for the baseline). The best score is highlighted in bold text. Although MouseGAN (Yu et al., 2022) utilizes an augmented dataset, it compromises intensity details, leading to the lowest precision and recall among all augmentation strategies. This performance gap largely stems from the fact that MouseGAN’s architecture is optimized for modality imputation in lower-resolution MRI. Its reliance on CycleGAN-like translation often results in ‘hallu-cinated’ or blurred textures when applied to high-resolution, synapsedense microscopy data. In contrast, a simpler mixing-based method like MixUp (Zhang et al., 2018) outperforms MouseGAN, despite introducing pixel-wise ambiguity. While CutMix (Yun et al., 2019) and CarveMix (Zhang et al., 2023) improve F1 and mIoU over the baseline, they fail to capture all true pixels, resulting in similar performance to MixUp. We hypothesize that this occurs because these methods generate synthetic samples without considering spatial locations, leading to overlapping regions that make distinguishing individual objects more challenging. By contrast, our proposed PRMix significantly outperforms all compared methods across all metrics, achieving the highest F1 (74.66) and mIoU (61.78) among all methods.

**Fig. 7:**
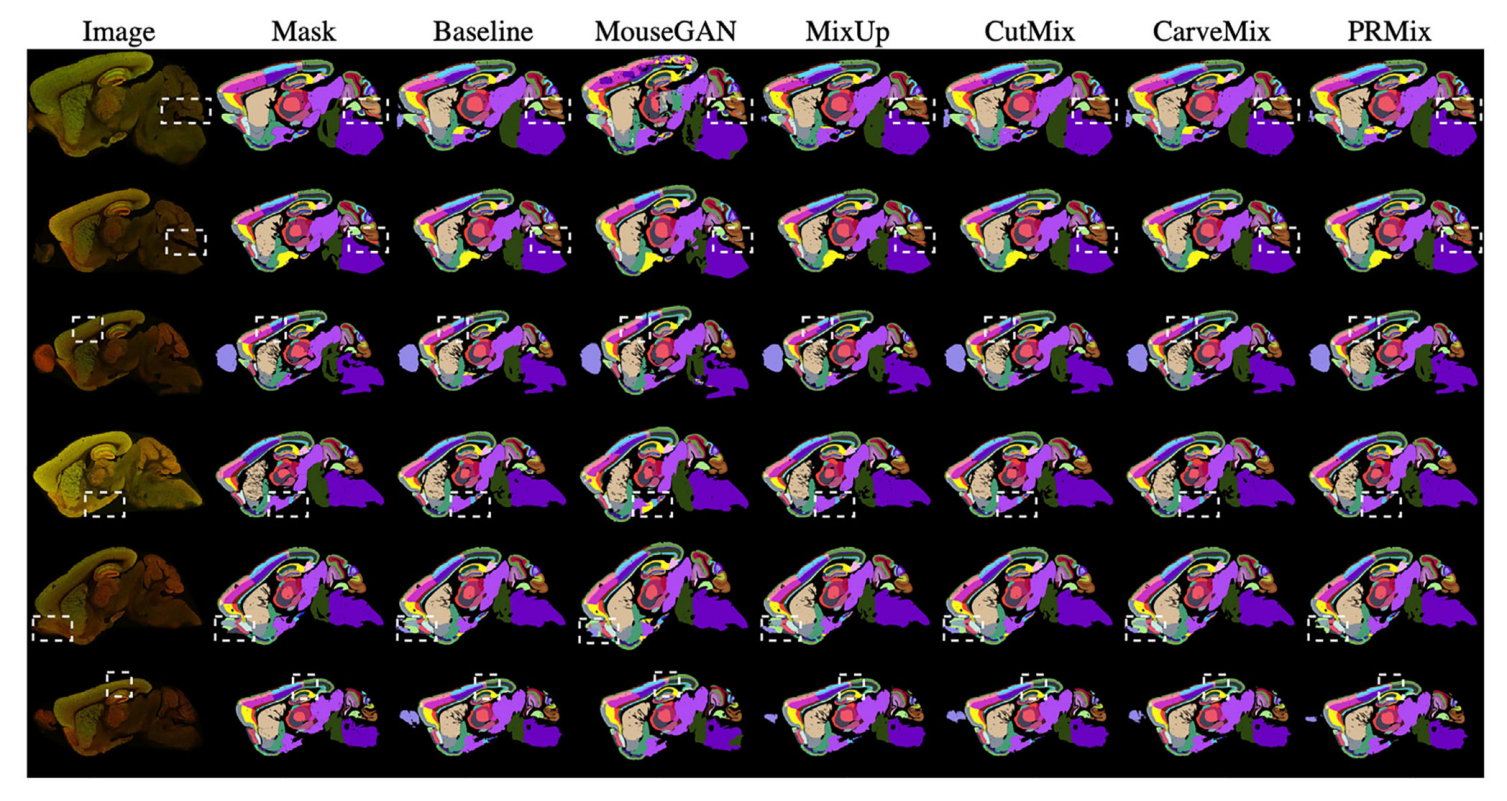
Visualization of results from different mixing methods on randomly selected testing samples. Dashed white rectangles indicate regions with the most significant discrepancies between methods. Best viewed digitally. Zoom-in images are available in the supplemental material.

Qualitative results from different methods are illustrated in Fig. 7. The top two rows highlight under-represented regions, such as UVUgr, which Baseline, MouseGAN, CutMix, and CarveMix fail to segment. These mispredictions can be attributed to insufficient training data (Baseline), severe overlaps from mixing (CutMix and CarveMix), and an inability to capture the intensity distribution (MouseGAN). The third row displays challenging cortical regions where most methods struggle. In contrast, our proposed PRMix produces results that align closely with the ground truth anatomy. The final three rows present highly ambiguous samples in the sAMY, AON, and PTLp regions. Here, MouseGAN’s result is particularly noisy due to a lack of intensity gradients, underscoring that both anatomical structure and intensity distribution are essential for accurate brain delineation.

To move beyond qualitative comparisons, which can be prone to selection bias, we conducted a rigorous statistical analysis. We performed a paired t-test on the F1 and mIoU scores for every test sample, comparing PRMix against each baseline and competing method. The results are detailed in Table 2. As expected, all data augmentation methods, including PRMix, significantly outperformed the baseline. More importantly, PRMix also showed statistically significant improvements over MouseGAN, MixUp, and CarveMix. Although the margin over CutMix was not statistically significant, the p-values for both F1 (0.079) and mIoU (0.057) were close to the significance threshold, and PRMix’s superior average performance is shown in Table 1.

### 5.3 Ablations

We conducted a comprehensive ablation study to evaluate the impact of several critical factors, including dataset scale, training duration, and the specific configuration of mixed images. Additionally, we investigated the influence of fluorescent channel selection, the proportion of hard samples in the HSM module, and the individual contributions of each PRMix component. Finally, we compared the effectiveness of purely synthetic training against a hybrid refinement schedule under a consistent computational budget, and all the results are detailed in Tables 3, 4, 5 and 6.

The results in Table 3(a) highlight the benefit of extensive data augmentation. Performance steadily improves as augmentation increases from 5× to 20×, where the model achieves its highest F1 and mIoU scores, surpassing the baseline by a large margin. Table 3(b) confirms that performance also correlates with longer training. However, comparing the two factors reveals that augmentation provides a greater advantage than training duration. For example, PRMix 20× with just 3 epochs of training outperforms PRMix 5× with 12 epochs, demonstrating that exposure to more diverse samples is more critical than longer training with less diverse data.

Our investigation into the number of mixed images (Table 4(a)) indicates that three is the optimal number. Mixing two images provides minimal variation and risks overfitting, whereas mixing four disrupts the target image’s structure. We attribute this to excessive structural disruption: when too many foreign patches are introduced, the global topological coherence of the target image is compromised. This results in an anatomically implausible ‘cluttered’ canvas where key contextual cues are heavily occluded, making it difficult for the model to learn valid spatial relationships. Empirically, mixing three images achieves the best balance and the highest performance. In Table 4(b), we assess the impact of fluorescence channels. The dual-channel model significantly outperforms models trained on either the SAP102 or PSD95 channel alone, validating our methodological design. We also note that the PSD95 model slightly outperforms the SAP102 model, likely because its clearer expression in maturer synapses (Metzbower et al., 2024) helps in distinguishing regional boundaries.

Table 5(a) shows that overemphasis on ‘easy’ samples (the 20% ‘hard’ setting) leads to suboptimal performance, likely due to insufficient feature diversity. In addition, the 50%:50% configuration does introduce greater diversity but still lacks enough hard examples to support learning a more generalizable representation, which accounts for its performance gap compared with the 80%–hard setting. Table 5(b) shows that integrating primary region sampling (PRS) alone yields a noticeable performance improvement compared to the baseline. However, further adding either HSM or OAA individually leads to performance degradation, suggesting that the ‘easy’ setting alone does not benefit from OAA, and that introducing additional hard samples through HSM increases task complexity. In contrast, combining both HSM and OAA produces a substantial performance gain, underscoring their synergistic effect in enhancing dataset quality.

For practical utility, we compared the ‘Purely Synthetic’ protocol (12 epochs) against a ‘Hybrid’ schedule (6 epochs synthetic pre-training + 6 epochs real data refinement) under a consistent compute budget. The results (Table 6) indicate that while incorporating real data yields marginal gains across all methods (0.2–1.2 increase in F1), PRMix maintains its consistent superiority. This confirms that PRMix generates high-quality synthetic samples that effectively complement real data in mixed training pipelines.

## 6 Conclusion

We curated the DMBM dataset, a high-resolution, expert-annotated collection capturing 118 brain subregions with unprecedented molecular and structural detail of the synaptic organization. For the first time, we demonstrate that integrating dual-fluorescence synaptic markers enables this dataset to serve as a comprehensive benchmark for regional delineation of the mouse brain.

To further improve the accuracy of automated segmentation methods, we introduced PRMix, a novel data augmentation that enables realistic data synthesis while preserving anatomical structures, achieving fine-grained brain region delineation beyond existing methods. By combining the DMBM dataset with PRMix augmentation, our work sets a new standard for mouse brain delineation and provides a robust framework for advancing neuroscience and biomedical imaging.

The resulting automated delineator offers a > 10-fold reduction in processing time, resolving boundary ambiguities and minimizing the inconsistencies of manual annotation. This efficiency dramatically lowers the barrier to creating large-scale, high-accuracy atlases. Looking forward, the adaptability of our framework opens critical new research avenues. While this study validated PRMix exclusively on the DMBM dataset due to the scarcity of comparable high-resolution synaptic data, the core principles of our approach — region-aware sampling and overlap minimization — are modality-agnostic. Consequently, our framework holds significant potential to generalize across diverse histological stains and species, providing a powerful tool for comparative neuroanatomy. Future work will focus on validating PRMix on external datasets and distinct imaging protocols to further establish its robustness. Ultimately, integrating this approach with other modalities, such as spatial transcriptomics and connectomics, will pave the way for a more holistic understanding of brain architecture.

## Supplementary Material

Supplementary material related to this article can be found online at https://doi.org/10.1016/j.neuroimage.2026.121881.

Supplementary material

## Figures and Tables

**Table 1:** Results on the isolated test set using different augmentation strategies, which are obtained with a 5-fold average. ±: standard deviations over 5 runs. Regional results are provided in the supplementary material.

Method	F1	mIoU	Precision	Recall
Baseline	65.33±0.49	54.33±0.48	66.35±0.87	66.55±0.40
MouseGAN 20×	70.62±0.28	57.11±0.15	71.45±0.46	71.35±0.37
MixUp 20×	73.30±0.53	60.73±0.4O	74.71±0.43	73.77±0.39
CutMix 20×	72.97±0.79	60.67±0.62	75.05±0.42	73.83±1.02
CarveMix 20×	73.30±0.85	60.87±0.56	74.10±1.07	74.44±0.49
PRMix 20×	**74.66+0.29**	**61.78±0.33**	**75.23±0.57**	**75.31±0.33**

**Table 2:** Paired t-test p-values for F1 and mIoU, computed on test samples. *P < 0.05, **P < 0.01 and ***P < 0.001. One-sided tests evaluate whether the selected method yields significantly higher F1 or mIoU than the reference. In each subgrid, the method above the horizontal line is the reference and the one below is the tested method.

Method	F1 Performance		mIoU Performance	
	F1-score	p-value	mIoU	p-value
Baseline	65.33	N/A	54.33	N/A
MouseGAN 20×	70.62**	**5.68e**−**3**	57.11*	**1.08e**−**2**
MixUp 20×	73.30***	**1.48e**−**17**	60.73***	**5.84e**−**21**
CutMix 20×	72.97***	**8.19e**−**22**	60.67***	**1.87e**−**24**
CarveMix 20×	73.30***	**7.21e**−**21**	60.87***	**1.87e**−**23**
PRMix 20×	74.66***	**1.72e**−**19**	61.78***	**8.69e**−**22**
MouseGAN 20×	70.62	N/A	57.11	N/A
PRMix 20×	74.66***	**4.78e**−**5**	61.78***	**2.897e**−**6**
MixUp 20×	73.30	N/A	60.73	N/A
PRMix 20×	74.66**	**9.32e**−**3**	61.78**	**7.83e**−**3**
CutMix 20×	72.97	N/A	60.67	N/A
PRMix 20×	74.66	7.89e−2	61.78	5.65e−2
CarveMix 20×	73.30	N/A	60.87	N/A
PRMix 20×	74.66	8.35e−2	61.78*	**4.61e**−**2**

**Table 3:** Ablation study on the impact of dataset scaling in augmented folds and training duration.

(a) Scaling effect				(b) The number of training epochs			
Method	Epochs	F1	mIoU	Method	Epochs	F1	mIoU
Baseline	12	65.33	54.33	Baseline	12	65.33	54.33
PRMix 5×	12	69.82	58.14	PRMix 20×	3	68.53	56.75
PRMix 10×	12	70.05	58.13	PRMix 20×	6	69.92	57.97
PRMix 20×	12	**74.66**	**61.78**		12	**74.66**	**61.78**

**Table 4:** Ablation on the number of mixing images and fluorescent channels.

(a) The number of mixing images			(b) Fluorescence channels		
Method	F1	mIoU	Method	F1	mIoU
Baseline	65.33	54.33	Baseline	65.33	54.33
PRMix Dual	74.34		PRMix_SAP102	69.92	57.77
PRMix Tri	**74.66**	**61.78**	PRMix_PSD95	70.00	58.44
PRMix Quad	71.03	59.50	PRMix	**74.66**	**61.78**

**Table 5:** Ablation on the portion of hard samples and key components.

(a) The portion of ‘hard’ samples in HSM			(b) Key components				
Method	F1	mIoU	PRS	HSM	OAA	F1	mIoU
Baseline	65.33	54.33	✓			73.70	60.76
PRMix 20%	72.73	59.98	✓	✓		71.17	59.21
PRMix 50%	69.71	58.02	✓		✓	70.60	58.57
PRMix 80%	**74.66**	**61.78**	✓	✓	✓	**74.66**	**61.78**

**Table 6:** Performance comparison of Synthetic (12 epochs) versus Hybrid (6 synthetic and 6 real epochs) strategies.

Method	Synthetic		Hybrid	
	F1	mIoU	F1	mIoU
MouseGAN 20×	70.62	57.11	71.01	57.51
MixUp 20×	73.30	60.73	73.73	61.10
CutMix 20×	72.97	60.67	73.73	61.01
CarveMix 20×	73.30	60.87	74.56	61.42
PRMix 20×	74.66	61.78	74.94	61.91

## Data Availability

Data and Code Availability Statement The data and code used for this study have been made public at https://git-pages.ecdf.ed.ac.uk/dmbm-datasets-5c13cd/. For the purpose of open access, the author has applied a CC-BY public copyright license to any Author Accepted Manuscript version arising from this submission.
