# Supplementary material for: PRMix: Primary Region Mix Augmentation and Benchmark Dataset for Precise Whole Mouse Brain Anatomical Delineation
Source: Neuroimage. Author manuscript; Available in PMC 2026 May 6. (PMC7619041; doi:10.1016/j.neuroimage.2026.121881)
Supplement: Supplementary material [file EMS213524-supplement-Supplementary_material.docx]

**Supplemental Materials**


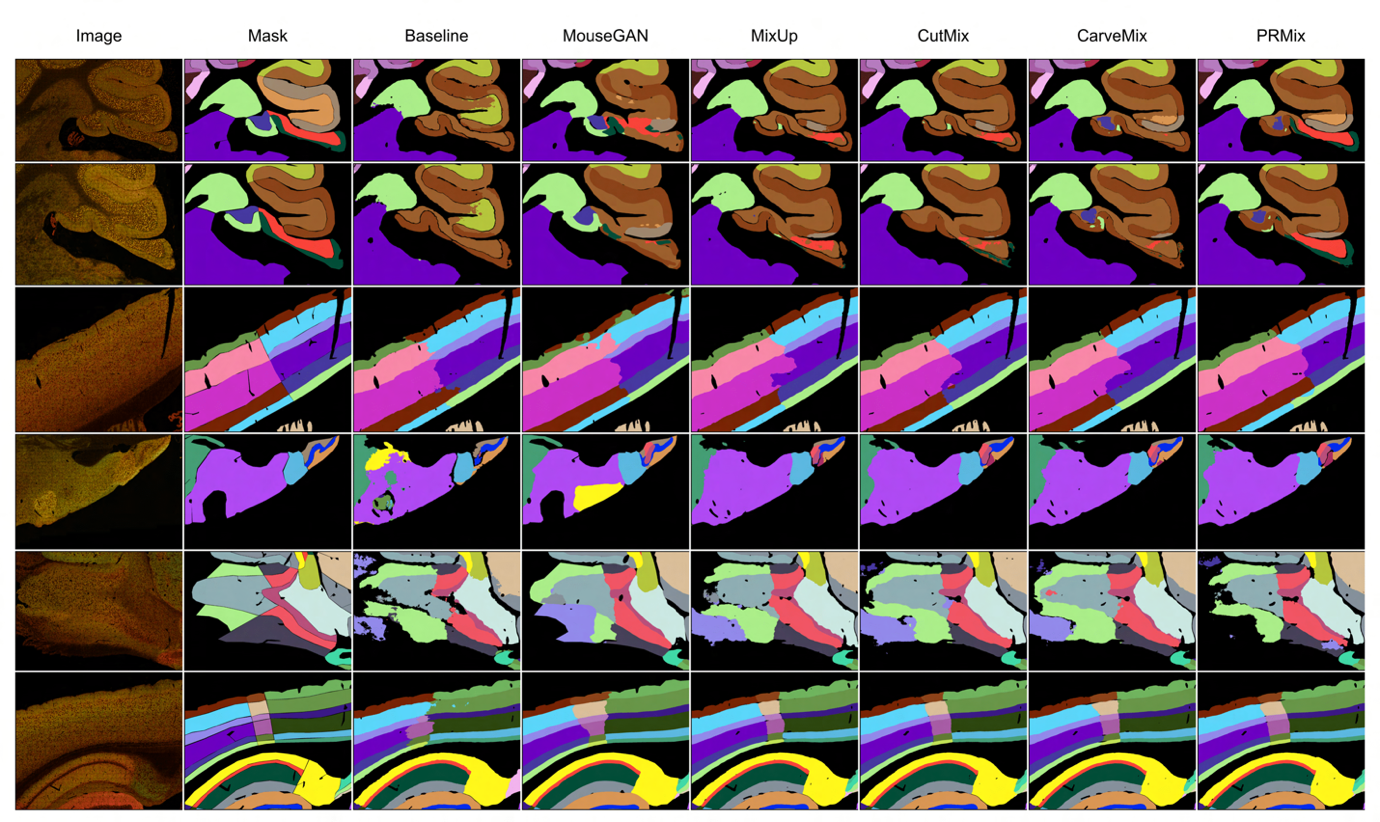


**Zoom-in visualization of boxed regions from Figure 7.** Discrepancies between the results obtained with different mixing methods on randomly selected testing samples are shown.


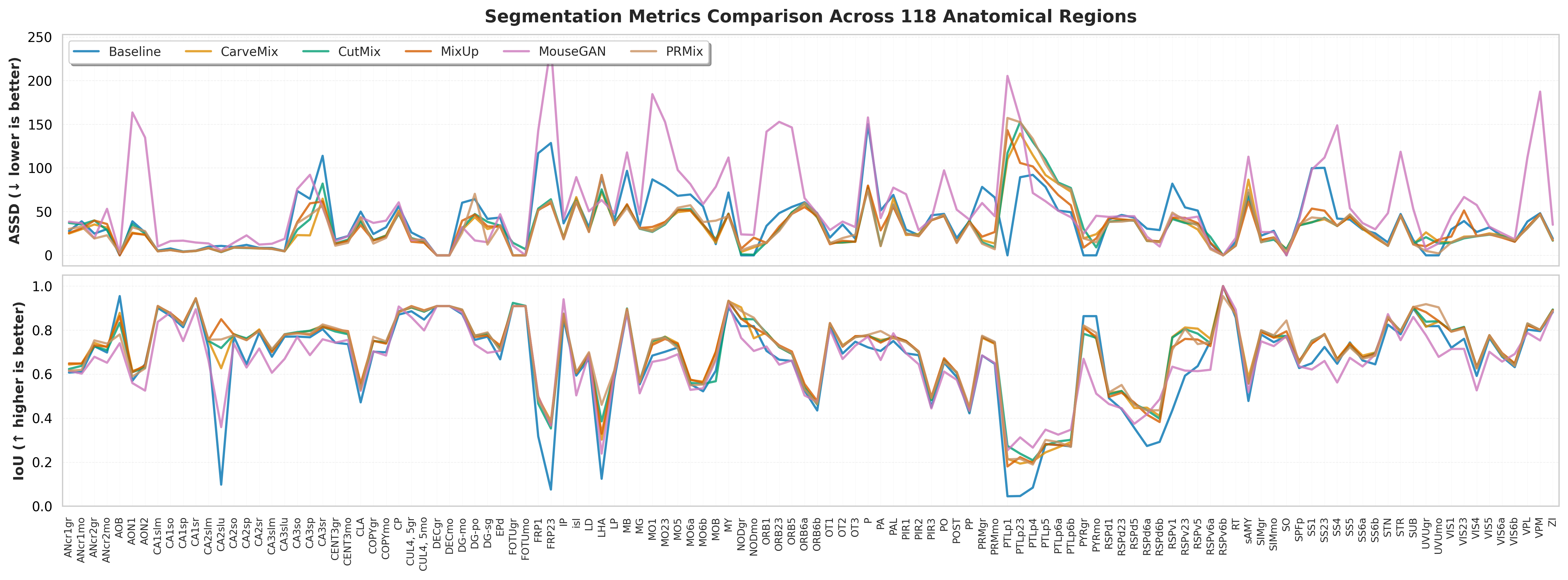


**Comparison of different augmentation strategies on 118 anatomical subregions of the mouse brain.** The top panel displays the Average Symmetric Surface Distance (ASSD), where lower values indicate better boundary adherence. The bottom panel displays the Intersection over Union (IoU), where higher values indicate better segmentation accuracy. The Baseline model exhibits significant performance volatility, characterized by sharp drops in IoU and spikes in ASSD for several challenging subregions. In contrast, PRMix consistently maintains superior boundary adherence (lower ASSD) and overlap (higher IoU) across the 118 anatomical regions. Notably, the generative method MouseGAN struggles with boundary precision—evidenced by substantially higher ASSD values across the board. This confirms that our region-aware mixing strategy not only improves global metrics but also specifically stabilizes segmentation performance for individual, anatomically distinct subregions.
